# Mapping interventional components and behavior change techniques used to promote self-management in people with multimorbidity: a scoping review

**DOI:** 10.1080/17437199.2023.2182813

**Published:** 2023-02-22

**Authors:** Madalina Jäger, Graziella Zangger, Alessio Bricca, Mette Dideriksen, Susan M. Smith, Julie Midtgaard, Rod S. Taylor, Søren T. Skou

**Affiliations:** a Research Unit for Musculoskeletal Function and Physiotherapy, Institute of Sports Science and Clinical Biomechanics, University of Southern Denmark, Odense, Denmark; b Danish Centre for Motivation and Behaviour Science, University of Southern Denmark, Odense, Denmark; c The Research Unit PROgrez, Department of Physiotherapy and Occupational Therapy, Næstved-Slagelse-Ringsted Hospitals, Slagelse, Denmark; d HRB Centre for Primary Care Research, Department of General Practice, Royal College of Surgeons in Ireland (RCSI), Dublin, Ireland; e Department of Clinical Medicine, University of Copenhagen, Copenhagen, Denmark; f Centre for Applied Research in Mental Health Care (CARMEN), Mental Health Centre Glostrup, Mental Health Services in the Capital Region of Denmark, University of Copenhagen, Copenhagen, Denmark; g MRC/CSO Social and Public Health Sciences Unit & Robertson Centre for Biostatistics, Institute of Health and Well Being, University of Glasgow, Glasgow, Scotland

**Keywords:** Multimorbidity, self-management, scoping review, behavior change techniques

## Abstract

Ageing populations and improved survival, have contributed to a rise in the number of people living with multimorbidity, raising issues related to polypharmacy, treatment burden, competing priorities and poor coordination of care. Self-management programs are increasingly included as an essential component of interventions to improve outcomes in this population. However, an overview of how interventions supporting self-management in patients with multimorbidity is missing. This scoping review focused on mapping the literature on patient-centered interventions for people living with multimorbidity. We searched several databases, clinical registries, and grey literature for RCTs published between 1990–2019 describing interventions that supported self-management in people with multimorbidity. We included 72 studies that were found to be very heterogeneous when it comes to the population, delivery modes and modalities, intervention elements and facilitators. The results pointed to an extensive use of cognitive behavioral therapy as a basis for interventions, as well as behavior change theories and disease management frameworks. The most coded behavior change techniques stemmed from the categories Social Support, Feedback and monitoring and Goals and Planning. To allow for implementation of effective interventions in clinical practice, improved reporting of intervention mechanisms in RCTs is warranted.

## Introduction

Multimorbidity, conceptualized as the co-existence of two or more chronic diseases in an individual is linked with decreased quality of life, functional decline, and increased healthcare utilization (Wallace et al., 2015).People living with multimorbidity commonly experience mental health difficulties, treatment burden, polypharmacy, and functional decline (Bayliss, Bayliss, Ware, & Steiner, 2004; Fortin et al., 2014; May, Montori, & Mair, 2009; Tinetti, Bogardus, & Agostini, 2004). This may have profound implications for research, treatment, and health policy (Fortin, Soubhi, Hudon, Bayliss, & Akker, 2007; Multimorbidity: A Priority for Global Health Research, 2018). Evidence highlights that treating one condition at a time is inconvenient, inefficient, and unsatisfactory for people with multimorbidity and their health care provider (Bower et al., 2011; Noël et al., 2007; Smith, O’Kelly, & O’Dowd, 2010). A more holistic approach is warranted that considers people’s social context, chronic conditions, and daily life (van der Aa, van den Broeke, Stronks, & Plochg, 2017).

A broad consensus is that multimorbidity is best addressed by a patient-centered approach (Multiple Chronic Conditions: A Strategic Framework, 2010., (Farmer et al., 2016). While there is no single definition of patient-centered care (or person-centered care), Bauman and colleagues regard it as the partnership between health practitioner and patient, based on communication and a focus that goes beyond specific conditions to emphasize health promotion and healthy lifestyles (Bauman et al., 2003). McWilliam highlighted that empowering patients to self-manage is central to patient-centered approaches (McWilliam, 2009). Chronic condition self-management and patient-centeredness are closely linked through the emphasis on shared responsibility and decision-making to achieve better health and wellbeing as defined by the person and the acknowledgement of the social, psychological, biological, and spiritual aspects that impact on self–management ability, placed within a context that respects the beliefs and values of the person (Lawn & Pols, 2005).

Self-management programs are increasingly recognized as an essential component of patientcentered interventions to improve outcomes and considered high-quality care for patients living with multimorbidity (Smith et al., 2016), (Bayliss et al., 2003). Some core elements for this population include providing education about multiple chronic conditions, psychological strategies to support the adjustment to life with chronic conditions, strategies to support adherence to multiple treatments, support around activities of daily living and physical functioning and providing social support (Taylor et al., 2014). There is a strong consensus that multiple causes of mortality and morbidity are linked with the behavior of individuals (Conner & Norman, 1996). Previous studies have emphasized the importance of unhealthy behavior (e.g., smoking, diet, alcohol use, physical inactivity) in the development and progression of multimorbidity (Wikström et al., 2015), (Fortin et al., 2014), (Dhalwani et al., 2016). Given the fact that behavior is modifiable, considerable efforts have been devoted to research, campaigns and interventions targeting health behaviors (e.g., weight control, smoking cessation, and increased physical activity) to improve health and thereby deter the development of chronic conditions and multimorbidity (Suls & Green, 2019). Defined as ‘overt behavioural patterns, actions and habits that relate to health maintenance, health restoration and health improvement’ (Gochman, 1997), health behaviors have received considerable attention from researchers, who explored the factors influencing how and why people engage in these behaviors (Conner et al., 2002). Understanding how (patient-centered) interventions might support selfmanagement in patients with multimorbidity is pivotal in promoting health in this population (e.g., by preventing or reversing physical inactivity). However, an overview of the literature in this area is missing.

A scoping review is ideally suited to determine the scope or coverage of a body of literature on a given topic as well as an overview of its volume and focus. In addition, it can be a useful tool to develop a theoretical understanding of the likely process of change by drawing on existing evidence and theory. This scoping review was part of the process of informing the development of an exercise and self-management intervention designed for people living with multimorbidity (see https://www.mobilize-project.dk/). Conducting a scoping review of the literature is a very good strategy to get an overview of the field.

Furthermore, despite the wide recognition of the biopsychosocial model and the connection between physical and psychological disorders, multimorbidity has not been a major focus in health psychology to date. This scoping review will be the first to contribute to a detailed understanding of the interventions evaluated in people with multiple chronic conditions by mapping the characteristics, types, duration, targets, delivery modes and modalities, impact, facilitators, behavior change techniques and frameworks/models used in the interventions. Classifying behavior change techniques used in interventions to promote self-management in people with multimorbidity will also be a first step in identifying potential mechanisms of change.

## Objectives

This scoping review focused on mapping the literature on patient-centered interventions for people living with multimorbidity that support self-management. In accordance with the definition of selfmanagement by Lorig and Holman (2003) (i.e., medical, or behavioral management of the disease, role management, and emotional management), we included a broad range of interventions focusing on health behavior change and/or providing psychological or social support or other approaches. These interventions were classified according to the underlying theory and behavior change techniques (BCTs) and strategies utilized.

## Materials and methods

This scoping review conformed to the framework for conducting scoping reviews developed by the Joanna Briggs Institute (Peters et al., 2015). The reporting followed the Preferred Reporting Items for Systematic reviews and Meta-Analyses extension for Scoping Reviews (PRISMA-ScR)(Tricco et al., 2018). The protocol for the scoping review was made public a priori and is available on the Open Science Framework (https://osf.io/eszb7).

## Eligibility criteria

The following eligibility criteria were formulated to identify relevant studies. The studies were randomized controlled trials published between January 2000 and November 2019 as multimorbidity is a newer term (Tugwell & Knottnerus, 2019). The participants were adults (18 + years old) living with multimorbidity, defined for this review, as having at least two of the following conditions: osteoarthritis (OA), ischemic heart disease or heart failure, hypertension, type 2 diabetes mellitus (T2DM), chronic obstructive pulmonary disease (COPD) and depression.^1^ Studies that targeted multimorbidity (two or more chronic conditions) where at least two of the conditions of interest for this scoping review were present were also included. Further, the interventions had to incorporate self-management elements directed at individuals. We focused on self-management elements that included patient education, support for decision-making, self-monitoring, and psychological and social support. We excluded interventions focusing solely on medical service usage (e.g., physician visits, hospital stays etc.) or merely evaluating professional and organizational interventions. In addition, one or more behavior change techniques (BCTs) had to be present for the study to be included. Studies focusing solely on medical service usage (e.g., physician visits, hospital stays etc.) or merely evaluating professional and organizational interventions were excluded. We did not apply any restriction to the type of comparator groups, outcome domains, country of origin or the language of the papers.

## Searching for the evidence

### Information sources

The search strategy (see Supplementary file 1) was adapted from two previous reviews (Bricca et al., 2020), (Willett et al., 2019). The final search strategy was tailored for use in the different databases.

The following electronic databases were searched: Cochrane Database of Systematic Reviews, Joanna Briggs Library, MEDLINE via PubMed, EMBASE via Ovid, CINAHL via Ovid, Scopus, and Psychinfo via Ovid. In addition, a search for grey literature was performed to identify unpublished trials.The search was done in the following databases: World Health Organization International Clinical Trials Registry Platform, Agency for Healthcare Research and Quality, ClinicalTrials.gov, Open-Grey.eu, and WorldCat.org. Citation tracking was be performed using Web of Science (WoS). Finally, the reference lists of reviews and trials found through the searches were hand-searched for additional references to ensure that relevant articles were not missed.

### Selecting the studies

The identified citations were uploaded to EndNote X9, and duplicates were removed. Due to the search size, four reviewers participated in the screening (MS, GZ, AB & MD). The identified studies were divided into two, and the two teams (AB & MD and MS & GZ) screened titles and abstracts independently and applied the eligibility criteria accordingly. Eligibility uncertainties were resolved by screening the full-text article and discuss until a consensus was reached. The qualified studies were then read in full text and evaluated against the eligibility criteria for the final decision by two reviewers (MS & GZ). Any disagreement was discussed until consensus. If information was missing (e.g., distribution of included conditions), the corresponding author was contacted via email. They were given two weeks to reply and sent a second email if they did not respond to the first one.

### Data charting process

Key variables were extracted using an a priori developed extraction template compiled in Microsoft Excel (2019) in duplicates by two reviewers (MJ & GZ). Study information on identifiers of the target population, purpose, context, intervention details, outcome(s), theoretical framework or theory, and the target behavior(s)/outcomes was extracted. The two reviewers checked the data and uncertainties were resolved by revisiting the full-text, supplementary materials or requesting additional information from the authors.

### BCT coding process

Each intervention was coded for BCTs using Michie et al. V1 behavior change taxonomy (Michie et al., 2013). BCTs have been conceptualized as replicable components of an intervention designed to alter or redirect causal processes that regulate behavior (e.g., *Social support*, *Providing feedback*, *Goal setting*, *Information about health consequences*). All the intervention elements (stated either in the manuscript, protocol, or supplementary material) that contained a specific BCT were coded. Two researchers (MJ & GZ) performed the coding independently after being trained in using the taxonomy. After coding the interventions, the two researchers discussed coding related issues and consulted a third researcher (ZS) for advice on these issues. Coding related disagreements were resolved through discussion. The final agreed coding was summarized in a grid presenting each study and the coded BCTs. The interventions were clustered by type, target behavior(s)/outcomes and combinations of chronic conditions they address. These elements were reported in the original studies or deduced from the content of the interventions. SPSS 26 (IBM SPSS Statistics for Windows [Internet], 2019) and Microsoft Excel (2019) were used to categorize and summarize the data. A table presenting the BCTs used in each study was developed. The total number of BCTs (individually and hierarchal groups) used across trials was calculated. A breakdown of the frequency and specific combinations of BCTs utilized in the interventions was compiled. The results were presented visually (using tables, charts, and diagrams) and in a descriptive format (narrative synthesis).

### Synthesis of results

The results are presented both in a visual form (e.g., using tables, and charts, as appropriate) and in a descriptive format (narrative synthesis) and include frequencies and proportions according to the intervention characteristics (type, duration, targets, delivery modes and modalities, impact, facilitators, behavior change techniques and frameworks/models utilized).

## Results

**Figure 1:**
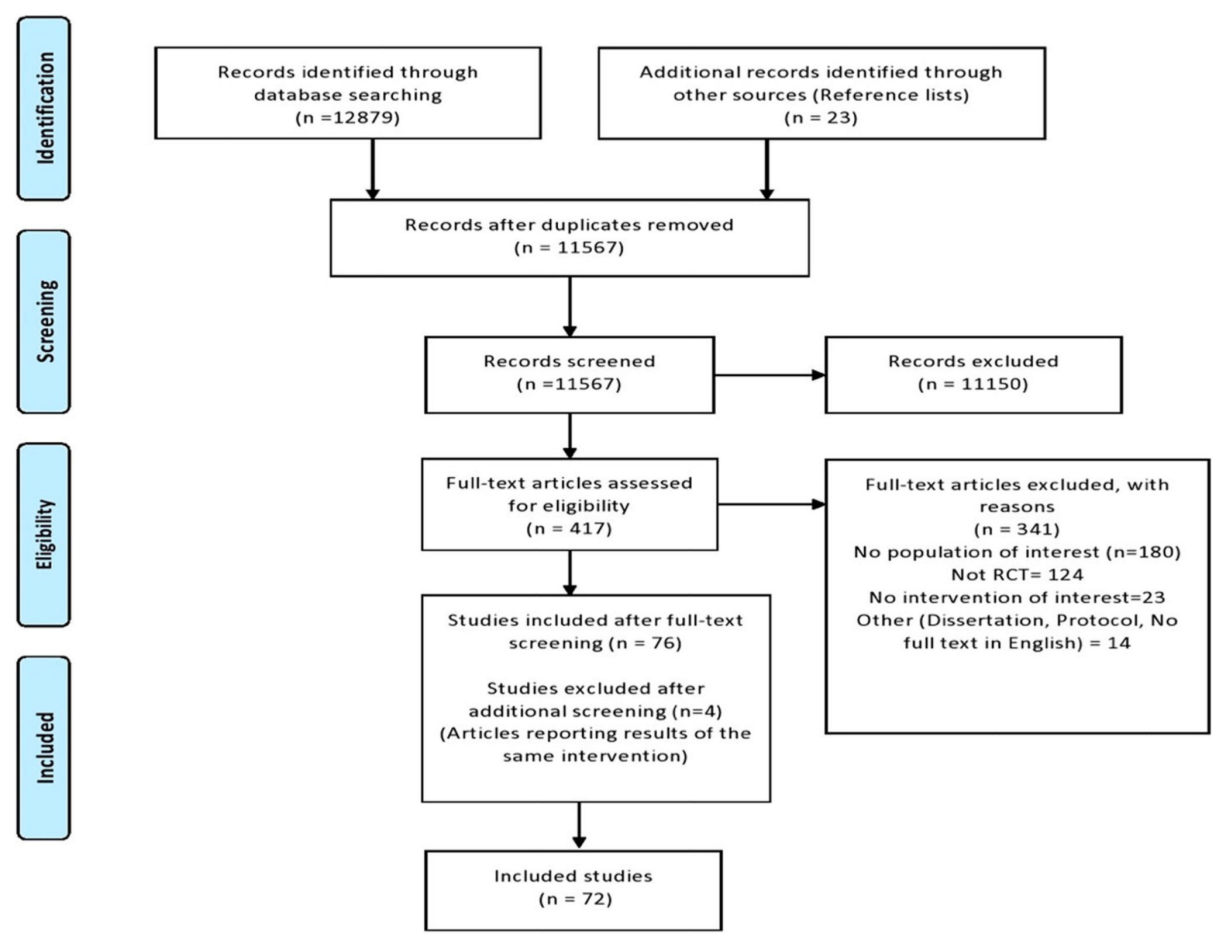
Prisma diagram illustrating the flow of information through the different phases.

The initial search yielded a total of 12,879 studies and 23 additional studies from other sources. After checking for full-text availability, removing duplicates and screening articles against the inclusion and exclusion criteria, a total of 72 RCT studies were identified for inclusion and data extraction (Figure 1). Table 1 presents the participants, intervention characteristics and target of interventions of the included RCT studies.

### Intervention characteristics

**Figure 2:**
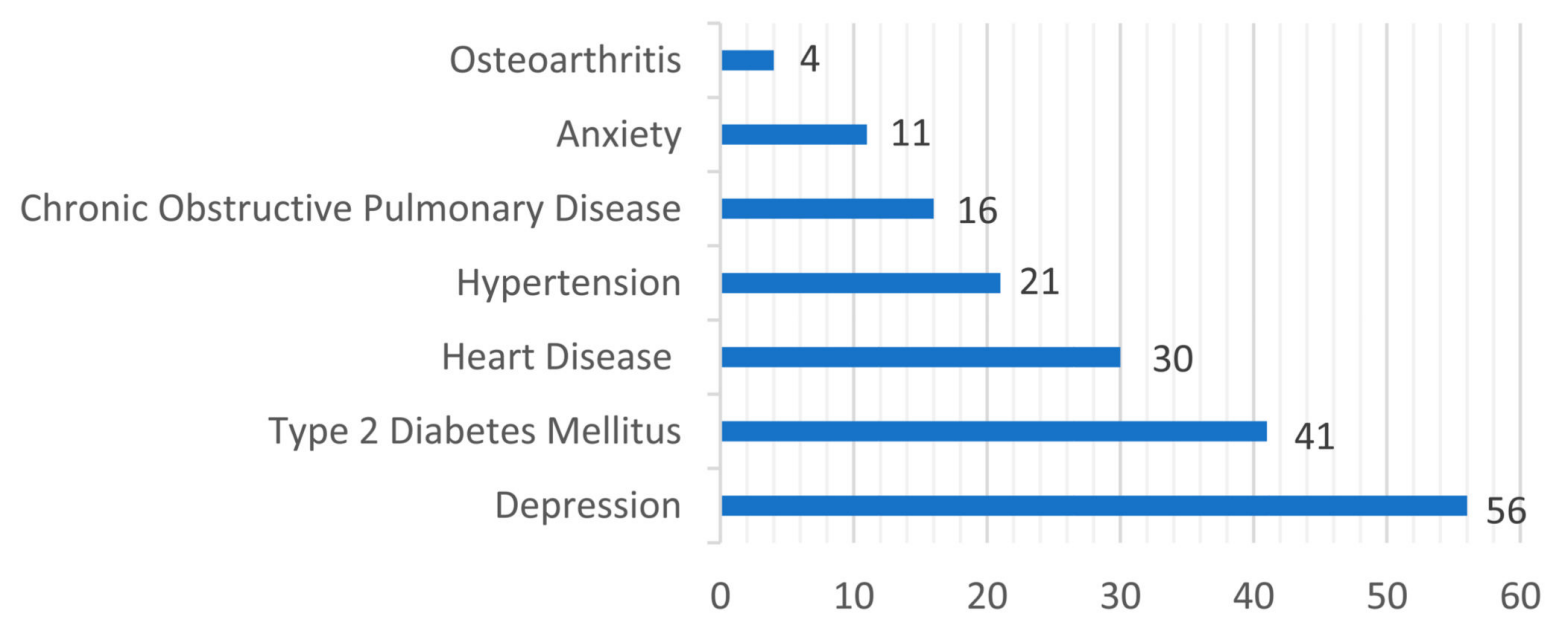
Presence of single conditions in the 72 studies.

**Figure 3:**
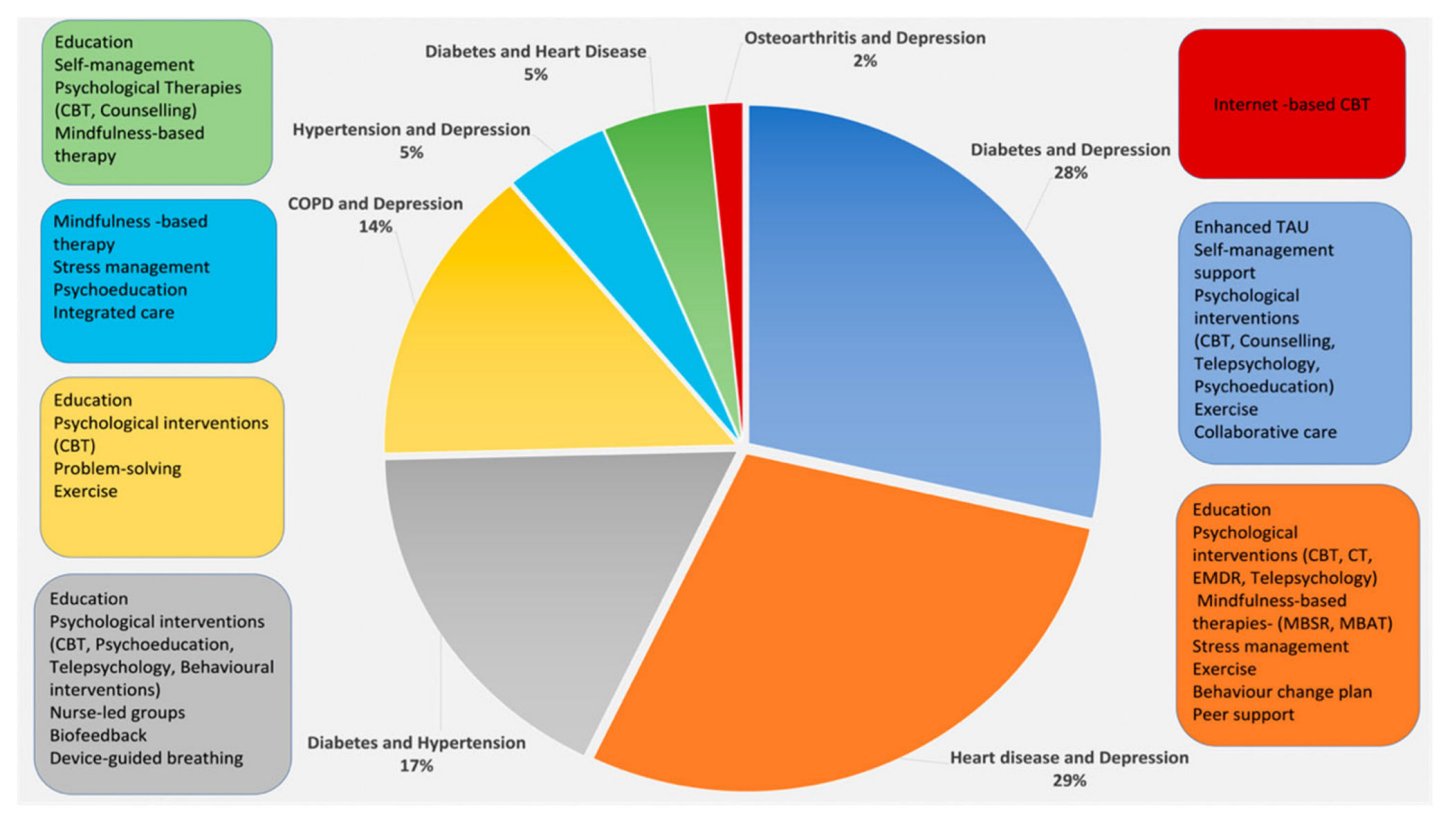
Types of interventions according to the most common clusters of conditions.

The 72 studies included a total of 13,866 participants. Age was giving in 65 of the 72 included studies, and the participants had a combined mean age of 61.1 years (SD 5.8). In seven studies, age was reported as a median or percentage, with a median age of 60.6 years. In the 70 studies that reported gender, 51% of the participants were females. In the 37 studies where ethnicity was stated, the majority was given as white (30%), other (10%), or African American (6%). The 72 studies were conducted in 20 different countries, with the USA being the most represented (44%), followed by Australia (10%), the United Kingdom (7%) and the Netherlands (7%). Depression was the most prevalent condition (56 studies), while osteoarthritis was the least occurring (4 studies). Regarding clusters of conditions, the most frequent combinations were heart disease and depression and Type 2 Diabetes and depression (both were present in 18 studies) (see Figure 3). Although not an inclusion criteri anxiety was present in 11 studies, either as an inclusion criterion for the single studies or as a comorbidity co-occurring with depression (see Figure 2).

### Intervention duration and target

Intervention duration was reported in 70 studies. In 41 of the studies (59%), the intervention duration was short (1-3 months), while 24% had a medium duration (4-6 months) and only 17% were of longer duration (6-12 months). Most interventions targeted depression (46 out of 72). Other interventions aimed to improve self-monitoring (18), self-care (14) and quality of life (14) and reduce anxiety (15).

### Intervention type and comparator

The 72 studies reported a diversity of interventions with different combinations of elements (see Figure 3). Most of the interventions (37) had a psychotherapeutic component (e.g., cognitive behavioral therapy (CBT) or internet-based CBT, Cognitive Therapy, Counseling, Mindfulness Therapy, Motivational Interviewing, Telepsychology) consistent with the fact that depression was the most prevalent single condition. Patient education, psychoeducation, self-management, and self-care were also frequently included (n = 31) in addition. Other elements less frequently encountered were music therapy, biofeedback, stress management and exercise therapy. We included studies utilizing different types of comparators, from usual care to active interventions. The most common comparators were usual care (n = 38) and enhanced usual care (n = 23).

### Delivery modes and modalities

Most of the interventions were delivered either face-to-face (n = 36) or both face-to-face and via telephone (n = 25). Only eight interventions had a web-based element, while two were delivered exclusively via telephone and one employed audiotape. Most of the interventions (n = 40) included were delivered one-to-one. Seventeen interventions were delivered in a group, ten were mixed, and five were self-guided.

### Primary outcomes and impact of interventions on outcomes

**Figure 4:**
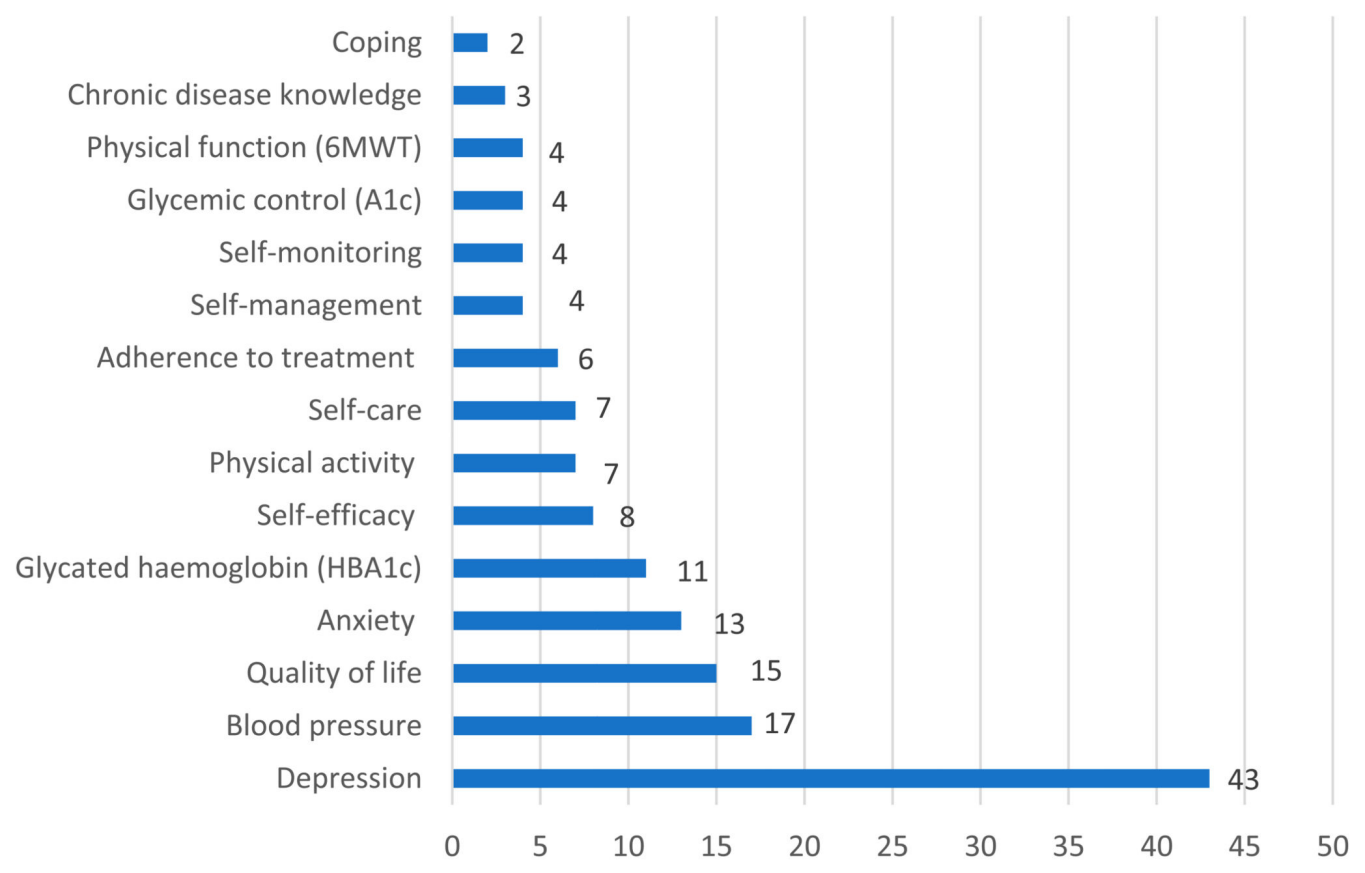
Frequency of primary outcomes.

The most frequently encountered primary outcome in the included studies was Depression (see Figure 4). Other commonly reported primary outcomes were blood pressure (17 studies), and quality of life (15 studies). Regarding intervention effectiveness (on primary outcomes), a large percentage of studies reported significant differences favoring the intervention group(s) (54 studies).

Sixteen studies reported that there was no significant difference in outcomes between the intervention and the control group, while two studies were reported as being underpowered.

### Professionals facilitating the interventions

Various professionals (i.e., diverse backgrounds and training) delivered the interventions either alone on their own or in a team. 28 interventions were delivered by nurses, followed by psychotherapists and psychologists (ten and eight, respectively). Other facilitators were physicians (n = 6), social workers (n = 3), occupational therapist (n = 1), pharmacists (n = 3), or physiotherapist (n = 1). Volunteers and research coordinators were also involved in the delivery of interventions.

### BCTs identified in the interventions

**Figure 5:**
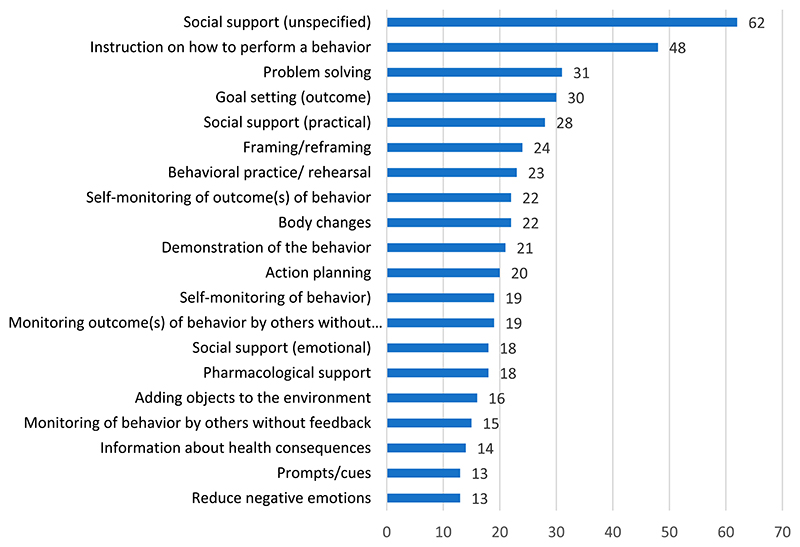
BCTs sorted by frequency.

A total of 582 different BCTs were coded ^2^ (see Supplementary file 2). The highest number of BCTs used in a single study was 16, while the lowest was two. The most frequently identified BCTs were *Social support (unspecified)* (coded 62 times), *Instruction on how to perform a behaviour* (n = 48), *Problem-solving* (n = 31) and *Goal setting (outcome)* (n = 30) (see Figure 5). In line with this, the clusters with the most coded BCTs were *Social support* (n = 108), *Feedback and monitoring* (n = 99) and *Goals and planning* (n = 96). The least coded BCTs were *Vicarious consequences*, *Self-talk*, *Rewarding completion*, *Valued self-identity* and *Incompatible beliefs* all coded one time. These BCTs correspond to the clusters: *Associations*, *Scheduled consequences*, *Self-belief*, *Covert learning* and *Reward and threat*.

Challenges arose when faced with coding BCTs present in interventions delivered to the comparator groups due to the lack of information describing their content. This was also because the most frequently encountered comparator was usual care or enhanced usual care, without providing details about usual care.

### Frameworks/models used in interventions

All 72 studies included either a framework, model, or theory to inform the development of the intervention (based on information from the articles and supplementary materials).

Specific information about how the theory/model was used was in most cases deemed insufficient or lacking.

## Discussion

To our knowledge, this is the first study to compile a comprehensive map of the interventional components and behavior change techniques used in patient-centered interventions that target self-management in people living with multimorbidity. We included 72 studies that reported interventions that were heterogeneous in terms of targeted populations, delivery modes and modalities, intervention elements, and facilitators (see Table 1 & Figure 5). The results pointed to extensive use of CBT as a basis for interventions, as well as behavior change theories and chronic disease management frameworks. In addition, the most coded BCTs stemmed from the categories of *Social Support*, *Feedback and monitoring*, and *Goals and planning*.

Very few studies have explored behavior change strategies utilized in patient-centered interventions for people living with chronic conditions. Findings from a previous review identifying BCTs and intervention features of dietary and physical activity interventions for patients with type 2 diabetes revealed that four BCTs were associated with a > 0.3% reduction in HbA1c: *Instruction on how to perform a behaviour, Behavioural practice/rehearsal, Demonstration of the behaviour and Action planning* (Cradock et al., 2017). However, contrary to our results that showed extensive use of theoretical models and frameworks as a basis for the interventions, this previous review by (Cradock et al., 2017) found that only three out of the thirteen RCTs included used a theory or model. Another similar systematic review focusing on the effects of BCTs for physical activity and healthy eating in overweight and obese adults found support for the use of goal setting and self-monitoring of behavior (Samdal et al., 2017). The authors of this review highlighted the importance of using a person-centered approach in maintaining healthy eating and physical activity long-term. The findings of our review are in line with previous literature focusing on the essential role of social support in improving outcomes for people with multimorbidity. Olaya and colleagues (Olaya et al., 2017) conducted a longitudinal survey including 2113 participants aged 60 + and confirmed the hypothesis that having two chronic physical conditions increased the risk of mortality over a 3-year follow-up period among people with low social support, compared with participants with no chronic illnesses. The authors emphasized that designing interventions that aim to increase social support can help improve the health status and survival of people who suffer from multimorbidity. Similarly, Vogel et al. explored the impact of perceived social support on health-related quality of life in people living with multimorbidity and found that higher perceived social support was associated with higher health-related quality of life scores (Vogel et al., 2012). Given the important role that social support (both emotional and practical) plays in the adaptation to chronic conditions (Eriksson & Rosenqvist, 1993), (King et al., 2018) future interventions designed for people experiencing multimorbidity should consider.

incorporating elements of social support (e.g., emotional support from family or friends, practical support from healthcare professionals). This may potentially act as a buffer to the limitations that multimorbidity imposes on people’s social lives (Sells et al., 2009). Finally, the findings of this review are in line with a Cochrane systematic review looking at interventions for improving outcomes in patients with multimorbidity in the primary care (Smith et al., 2021) in those interventions designed for this population are complex and multifaceted, reflecting the different needs of the individuals.

### Limitations

One limitation is related to the poor reporting and vague description of interventions, which contributed to difficulties in coding the BCTs utilized in the studies. Furthermore, we cannot judge the quality of the trials included due to the absence of a quality assessment, which was beyond the scope of this review, that aimed to map the literature on interventions in this specific area. Another important limitation is the short duration of interventions, most of which (60%) were short (1-3 months). At best, the studies provide an indication of the characteristics of short-term interventions supporting self-management, lacking a focus on the maintenance of behavior change which is fundamental in self-management long-term.

While we included studies published until 2019 we believe that given the large number of studies included (k = 72) and the level of details of our analyses, including the BCT assessment, our results provide an unbiased and comprehensive overview of the topic.

Finally, defining multimorbidity as living with two or more of six specific chronic conditions as well as the period searched (2000-2019) may have limited the volume of literature selected for this review.

### Implications

The findings presented here provide a starting point for further investigation of patient-centered interventions that include self-management designed for people living with multiple chronic conditions. Within the context of multimorbidity, there are some populations or clusters that could benefit from more focus. For example, while a vast volume of research has concentrated on people living with diabetes and depression or heart disease and depression, only a restricted number of trials were designed for people living with multimorbidity involving COPD or osteoarthritis.

There is a need for a better understanding of the emergence and persistence of multiple chronic conditions as well as an awareness of the relationship between psychological, behavioral, social, and environmental factors and multiple chronic conditions. Despite that the focus of health psychology is studying the connection between physical and psychological disorders, including the psychological determinants of health behavior, multimorbidity has not been a primary concern (Suls & Green, 2019). Nevertheless, health psychology has demonstrated the adoption of a biopsychosocial approach concerning the co-occurrence of psychological and medical conditions. Interventions are needed to promote health behavior adherence among people with chronic conditions that also address the challenges of living with multimorbidity. Recent developments in the design of health behavior interventions have highlighted the importance of theory and classifying intervention components (BCTs) (Michie et al., 2013) and mapping these intervention components onto mechanisms of change. Clear and accurate reporting of interventions is needed (Michie et al., 2009). Both CONSORT and TIDieR guidelines are useful in providing recommendations for reporting specific characteristics such as intervention content and delivery (Moher et al., 2001), (Hoffmann et al., 2014). To further develop effective programs for people with multiple chronic conditions, researchers should adhere to MRC guidelines by incorporating behavior change theory into interventions (Craig et al., 2008) and adequately report the relevant active components or BCTs (Painter et al., 2008). This will contribute to increased transparency and improved reporting as well as provide clinicians with sufficient information to implement effective interventions, also facilitating the understanding of potential intervention mechanisms.

We suggest that future research should focus on enriching the existing evidence base regarding the effectiveness of interventions for people living with multimorbidity. This aligns well with Smith et al. (2021) who highlighted that there are remaining gaps in our knowledge about the effectiveness of interventions for people with multimorbidity despite the increased number of RCTs in this area. One of the challenges is that the interventions tested so far for people with multimorbidity were not developed and tailored to this population (but for people with single chronic conditions). This might have contributed to the small effects observed in systematic reviews (Smith et al., 2021). Moreover, in addition to effectiveness, it is also important to consider the potential for implementation of interventions and the contextual factors relevant in their implementation (MRC Framework, 2021).

Finally, intervention designers should consider a full range of different components when developing patient-centered programs for people living with multimorbidity, using previous research evidence and recommendations, and based on a good understanding of patients’ needs and preferences, preferably also involving these when designing interventions. Not only will this enhance the acceptability of the interventions, but it will potentially lead to higher effectiveness.

## Conclusions

This review highlighted the complexity and diversity of patient-centered interventions designed for people living with multiple chronic conditions. Even though all the interventions included in this review were informed either by a chronic disease management framework, psychotherapeutic approach, behavior change model, or theory, reporting needs to improve to allow adequate coding of BCTs and evaluation and implementation of effective interventions in clinical practice.

## Figures and Tables

**Table 1:** Characteristics of included studies.

Author, Year, Country	Participants	Type of intervention	Target of intervention	Comparator^3^	Theory mentioned in text
(Ahmadpanah et al., 2016), Iran	Hypertension & Depression N = 45	Metacognitive detached mindfulness therapy (MDM) or Stress management training (SMT)	Reducing high blood pressure, depression, and anxiety	UC +	Metacognitive detached mindfulness therapy (MDM), stress management principles (SMT), systematic desensitization (Wolpe 1958) attention training techniques (ATT) (Kang, Gruber, & Gray, 2013)
(Alexopoulos et al., 2013) United States	COPD & Depression N = 138	Personalised intervention for depression and COPD (PID-C)	Depression, adherence to rehabilitation and treatment	UC	Theory of reasoned action (TRA)
(Alexopoulos et al., 2016), United States	COPD & Depression N = 101	Problem Solving-Adherence (PSA) and personalized intervention (PID-C)	Depression, adherence to rehabilitation and treatment	PID-C vs. PSA	Problem- solving therapy (PST)
(Barker et al., 2018), Australia	MM (COPD, bronchiectasis, heart failure, coronary artery disease or ischemic heart disease and at least one other chronic condition, such as diabetes, hypertension, and cancer) N = 17	A multimorbidity rehabilitation program (exercise and education)	Improving exercise capacity, self-management, quality of life and reducing hospitalizations	UC	Multimorbidity rehabilitation
(Barley et al., 2014), United Kingdom	Heart Disease & Depression N = 81	Personalised care (PC)	Improving Selfmanagement, reducing depression	UC	Self-efficacy (goal setting and action planning)
(Beckie et al., 2011), United Kingdom	Heart Disease & Depression N = 252	Behavior change plan (Informed by TTM)	Reducing depression	UC	Transtheoretical model (TTM) and Motivational Interviewing (MI)
(Behnammoghadam et al., 2015), Iran	Heart Disease & Depression N = 60	EMDR therapy	Reducing depression	UC +	EMDR therapy
(Berkman et al., 2003) United States	Heart Disease & Depression N = 2481	Psychoeducation and medication	Reducing depression and increasing social support	UC +	CBT, Social Learning Theory
(Bogner & de Vries, 2008), United States	Hypertension & Depression N =64	Integrated care	Improving adherence to medication, increase BP control, and reduce depression	UC	Theory of reasoned action (TRA)
(Bogner & de Vries, 2010), United States	Diabetes & Depression N = 58	Integrated care	Improve medication adherence, glycaemic control and reduce depression	UC	Theory of reasoned action (TRA)
(Bogner et al., 2012), United States	Diabetes & Depression N = 180	Integrated care	Improve adherence to medication, glycaemic control and depression.	UC	Theory of reasoned action (TRA)
(Bogner et al., 2013) United States	Hypertension & Depression N =60	Integrated care	Improve BP control and depressive symptoms.	UC	Theory of reasoned action (TRA)
	Diabetes & Depression N = 360	Self-management support		UC +	Self-management support
(Cherrington et al., 2018), United States			Improving self-management (T2DM control, exercise, diet, stress management), reducing depressive symptoms and healthcare utilization)		
(Clarke et al., 2019), Australia	Diabetes & Depression N = 723	Self-guided CBT	Improving work and social functioning (in T2DM), reducing depression	CG + Placebo control program (Healthy Lifestyles) with health and lifestyle information	CBT
(Coventry et al., 2015), United Kingdom	MM (diabetes and/or coronary heart disease, Depression) N = 387	Collaborative care	Reduce depressive symptoms and anxiety, increase self-management	UC	Collaborative care model, ABC model
(de Godoy & de Godoy, 2003), Brazil	COPD & Depression N = 30	Exercise, psychotherapy and education	Reduce depression and anxiety	CG + PT, Exercise, Education	Exercise, psychotherapy, and education
(de Groot et al., 2019), United States	Diabetes & Depression N = 140	Psychotherapy, exercise, and education	Reduce depression	UC or EX (Exercise only) or CBT + EX all offered the Dining with Diabetes nutrition education program	Beck’s model of cognitive therapy
(Dejesus et al., 2009) United States	Diabetes & Hypertension N = 54	Education and home BP selfmonitoring	Improve self-management	UC	Chronic disease model
(Dekker et al., 2012), United States	Heart Disease & Depression N = 41	Brief cognitive therapy	Reduce depressive symptoms and negative thinking, improve QoL	UC	Beck’s model of cognitive therapy (CT)
(Denver et al., 2003), United Kingdom	Diabetes & Hypertension N = 120	Nurse-led HT clinic group	Improve management of uncontrolled HT	UC	Nurse-led HT clinic group
(Doyle et al., 2017), Australia	COPD & Depression N=110	Telephone CBT	Reducing depression and anxiety and improving self-efficacy	UC + Active social control (befriending)	CBT, Befriending
(Dunbar et al., 2014), United States	Diabetes & Heart Disease N = 65	Education, counseling, selfmanagement	Self-care and selfmanagement (diet, medication, symptom monitoring, physical activity, and recognition of the interaction between self-management strategies for HF and DM)	UC +	Integrated theoretical framework to guide HF and DM self-care
(Dunbar et al., 2014), United States	Diabetes & Heart Disease N = 134	Integrated HF-T2DM Self-Care	Integrate and improve HF and DM self-care (diet, medications, selfmonitoring, symptoms, and PA)	UC +	Integrated theoretical framework to guide HF and DM self-care
(Edelman et al., 2010) United States	Diabetes & Hypertension N = 239	Self-management and medical clinic education groups	Improve self-care and selfmanagement	UC	Self-management model, Group medical clinics
(Edelman et al., 2015), United States	Diabetes & Hypertension N = 377	Tailored behavioral intervention	Improve self-management of comorbid T2DM and HT	UC +	Nurse led behavioral management
(Egede et al., 2018), United States	Diabetes & Depression N = 90	Reducing depression and anxiety and improving glycaemic control	Reducing depression and anxiety and improving glycaemic control	BAT via telemed at home vs. BAT in same room	Behavioral activation
(Fortin et al., 2016), Canada	MM (Diabetes, cardiovascular disease, COPD, asthma, tobacco smoking, obesity, hyperlipidaemia, Prediabetes) N = 332	Self-management support and health education	Improve chronic disease prevention and selfmanagement of multimorbidity	CG + 3 months delayed intervention	Chronic disease prevention and management (CDPM)
(Freedland et al., 2009), United States	Heart Disease & Depression N = 123	CBT or Supportive stress management and medication	Reducing depression and improving HF self-care after surgery	UC	Cognitive behavior therapy (CBT), Stress management
(Freedland et al., 2015), United States	Heart Disease & Depression N = 158	CBT and tailored education (for HF)	Reduce depression and improve self-care in HF patients	UC	CBT and tailored education (for HF)
(Gary et al., 2010), United States	Heart Disease & Depression N = 74	CBT, exercise, CBT and exercise	Improving physical activity, reducing depression, and enhancing quality of life	CBT + Exercise vs CBT only vs Exercise only vs. UC	CBT (Beck’s model of depression)
(Garvey et al., 2015), Ireland	MM (two or more chronic conditions, 43 conditions identified, most common: arthritis, congestive cardiac failure, diabetes, depression and hypertension) N = 50	Multimodal program: Fatigue management; healthy eating; maintaining physical activity, maintaining mental health; managing medications and communicating effectively with health professional	Improving activity participation, self-efficacy, self-management and quality of life	UC + Waiting list (invited to attend an OPTIMAL course following trial completion)	Adapted Stanford Chronic Disease Self-Management Program, Bandura’s model of Self efficacy
(Glasgow et al., 2006), United States	Diabetes & Depression N = 335	Computer-assisted Selfmanagement Intervention (focused on healthy eating and physical activity)	Improving diabetes selfmanagement (by improving healthy eating and exercise)	UC	Chronic care model selfmanagement framework, Motivational interviewing (MI) (Miller & Rollnick)
(Hochhalter et al., 2010), United States	MM (treated for at least two of following: arthritis, lung disease, heart disease, diabetes, hypertension, depression, and osteoporosis) N = 79	Patient engagement intervention or safety Group	Improve patients’ selfefficacy for managing multimorbidity	Group A: Intervention (Appointment - workshop + phone call) Group B: Safety (attention control - workshop + phone call) Group C: UC	Self-Determination Theory (SDT)
(Huang et al., 2016), Taiwan	Diabetes & Depression N = 61	Motivational enhancement therapy (MET) and CBT	Reduce depressive symptoms and increase quality of life	UC +	Theory of stress and coping (Lazarus and Folkman) modified by Miller & Rollnick’s motivational principles
(Hynninen et al., 2010), Norway	COPD & Depression N = 51	CBT	Reduce anxiety and depression	UC +	CBT adapted by Stanley et al.)
(Jang et al., 2018), Korea	Heart Disease & Depression N =44	Mindfulness-based art therapy (MBAT)	Reduce depression, trait anxiety, anger and anger expression	UC [Waiting list control (received MBAT afterwards)]	Kabat-Zinn’s mindfulness meditation (MBSR,) Monti’s Mindfulness-based art therapy (MBAT) and Selfregulation theory
(Kasteleyn et al.,2016), Netherlands	Diabetes & Heart Disease N = 161	Self-management support, motivational interviewing	Improve self-management, self-efficacy and wellbeing, reducing distress	UC + Attention control, 1 telephone consultation	Leventhal’s Common-Sense Model of self-regulation (CSM), Bandura’s Social Cognitive Theory, Motivational interviewing
(Kunik et al., 2008), United States	COPD & Depression N = 238	CBT	Reduce anxiety and depression	Education group	CBT
(Lamers et al., 2010), Netherlands	COPD & Depression N = 187	Minimal Psychological Intervention (MPI)	Reducing depression and anxiety, and improving quality of life	UC	CBT and self-management
(Lamers et al., 2011), Netherlands	Diabetes & Depression N = 208	Minimal Psychological Intervention (MPI)	Reducing depression and improving quality of life	UC	CBT and self-management
(Landman et al.,2013), Netherlands	Diabetes & Hypertension N = 48	Biofeedback	Lowering blood pressure	CG [visually identical device guiding users to a breathing frequency of approximately 14 breaths/ min]	Biofeedback
(Liu et al.,2019), China	COPD & Depression N = 60	Reduce depression and improve the quality of life	Reduce depression and improve the quality of life	UC +	Music Therapy
(Logan et al., 2012), Canada	Diabetes & Hypertension N = 110	Telemonitoring Self-Care Support System	Improve blood pressure control and self-care	UC	Telemonitoring Self-Care Support System
(Logtenberg et al., 2007), Netherlands	Diabetes & Hypertension N = 30	Device-guided breathing (Resperate)	Change in mean daytime ambulatory BP	UC +	Device-guided breathing (Resperate)
(Long et al., 2015), China	Diabetes & Depression N = 100	Group counseling	Reduce depression, improve treatment compliance and blood sugar control	UC +	Cognitive-behavioral group counseling, group dynamics
(Ludman et al., 2013), United States	MM (Depression, Diabetes and/or coronary heart disease) N = 214	TEAM care (self-management support, monitoring disease and pharmacotherapy)	Improving self-care-efficacy and confidence to maintain lifestyle changes (diet and exercise), reducing depression	UC	Collaborative care, Social Cognitive Theory, Self-management support
(Lundgren et al., 2016), Sweden	Heart Disease & Depression N = 50	Internet-Based Cognitive Behavioral Therapy (ICBT)	Reduce depression and anxiety and improve quality of life	CG +	CBT
(Lutes et al., 2018), United States	Diabetes & Depression N = 139	Collaborative care	Reducing depression and diabetes distress, improving self-care (diet, physical activity, smoking, foot care)	UC + educational materials	CBT, Problem-solving Therapy (PST), Collaborative care model
(Lynch et al., 2014), United States	Diabetes & Hypertension N = 61	Self-management intervention-Lifestyle Improvement through Food and Exercise (LIFE)	Improve self-management (diet, physical activity, and glycaemic control)	CG	Information processing model for food choice, self-management
(Moral et al., 2015), Spain	MM (mean of 5 chronic conditions/patient: hypertension and diabetes -the most prevalent) N = 154	Motivational Interviewing	To promote adherence to treatment in people treated by polypharmacy	UC	Motivational Interviewing (MI)
(Nie et al., 2019) China	Heart Disease & Depression N = 284	Education and lifestyle improving program (IPEL)	Reducing anxiety and depression, improving lifestyle (healthy diet, physical activity, and smoking cessation)	UC	Multicare intervention
(Norlund et al., 2018), Sweden	Heart Disease & Depression N = 239	Internet-Based Cognitive Behavioral Therapy (ICBT)	Reducing depression and anxiety	TAU	CBT
(O’Moore et al., 2018), Australia	Osteoarthritis & Depression N = 69	ICBT Sadness Program	Reducing depression	UC	CBT
(O’Neil et al., 2014), Australia	Heart Disease & Depression N = 121	Reducing depression, improving self-management	Reducing depression, improving selfmanagement	UC	CBT
(Onyechi et al., 2016), Nigeria	Diabetes & Depression N = 80	Cognitive behavioral coaching	Reducing depression	UC	Cognitive Behavioral Coaching for Depression Manual (CBCDM)
(Ose et al., 2019), Germany	MM (Diabetes and at least two other severe chronic comorbidities) N = 495	Care management program	Improving self-management and self-care (diet, exercise, self-monitoring of blood glucose and foot care)	UC	Care management, Primary Care Network (PCN)
(Parswani et al., 2013), New Zealand	Heart Disease & Depression N =30	MBSR	Reduce anxiety and depression	TAU + health education session	Mindfulness meditation (Jon Kabat-Zinn, Segal et al.)
(Penckofer et al., 2012), United States	Diabetes & Depression N = 74	Psychoeducation (SWEEP program) CBT and education	Reduce depression, anxiety and anger and improve quality of life	UC +	CBT, ‘Reality Management Approach for Persons with Depression’ (Munoz et al.)
(Pibernik-Okanovic et al., 2015), Croatia	Diabetes & Depression N = 209	Psychoeducation, Physical exercise, enhanced UC	Reducing depression, improving diabetes distress, self-management and quality of life	Group A: CBT + Self-help manual. Group B: Exercise (same interval and duration as CBT) Group C: Enhanced treatment as usual	CBT
	Diabetes & Depression N = 291			UC +	CBT
(Piette et al., 2011), United States		Telephone-delivered CBT program	Improve management of depressive symptoms, physical activity levels, and diabetes-related outcomes		
(Rachmani et al., 2002), Israel	Diabetes & Hypertension N = 141	Patient Participation Program	Improve self-management, self-monitoring, and disease control	UC	Intensive therapy, self-management
(Renn et al., 2018), United States	COPD & Depression N=175	Brief CBT (Psychoeducation, chronic disease selfmanagement, goal setting, behavioral activation, cognitive restructuring, relaxation, and coping with physical health symptoms)	Reduce illness intrusiveness and improve selfmanagement	EUC (enhanced usual care)	CBT
(Richards et al., 2018), United Kingdom	Heart Disease & Depression N = 29	EPC (enhanced psychological care)	Reduce depression and anxiety, mortality and morbidity and improve quality of life	UC	Enhanced psychological care, rehabilitation
(Safren et al., 2014), United States	Diabetes & Depression N = 87	Enhanced treatment as usual, CBT	Reduce depression, improve adherence and glycaemic control	UC +	CBT, Motivational interviewing (MI)
(Salisbury et al., 2018), United Kingdom	MM (at least three types of chronic condition) N = 1546	Patient-centred care model (3D approach)	Improve patient-cantered care, self-care, and quality of life	UC	Patient-centred care model (3D approach)
(Schneider et al., 2016), United States	Diabetes & Depression N = 29	Behavioral Activation and Exercise	Increasing exercise level and exercise enjoyment	Enhanced Usual Care + Depression treatment referrals were also provided. Participants received information available from the ADA on nutrition, exercise, and glucose monitoring	Behavioral Activation (BA)
(Shen et al., 2018), China	Heart Disease & Depression N = 60	Psychological Intervention Program	Improving mental state, reducing stress and negative coping styles	UC	Comprehensive psychological intervention (cognitive therapy; relaxation therapy, emotional support)
(Taveira et al., 2011), United States	Diabetes & Depression N = 88	Psychoeducation (Pharmacist- Led Group Medical Appointments), Shared medical appointments (SMAs)	Reducing depression, improving self-care and self-care competence	UC	Psychoeducation (Pharmacist-Led Group Medical Appointments), Shared medical appointments (SMAs)
		ICBT		UC	
(Turner et al., 2014), Australia	Heart Disease & Depression N = 42		Decreasing depression, increasing self- efficacy, improve health perceptions and behavior change (physical activity, diet, medication adherence, and smoking cessation)		CBT and motivational interviewing (MI)
(Wakefield et al., 2011), United States	Diabetes & Hypertension N = 302	Home telehealth device and nurse care management	Improve self-care (diet, exercise, smoking cessation, foot care, advice for sick days, medication, weight management, preventive care)	High-intensity vs low- intensity vs UC	Care management
(Wu et al., 2012) Australia	Heart Disease & Depression N = 30	Peer CDSMP (self-management)	Improving knowledge, selfefficacy, and self-care behavior	UC +	Bandura’s theory of selfmanagement (Bandura, 1977, 2004)
(Yoo et al., 2009), Korea, Republic of	Diabetes & Hypertension N = 123	Ubiquitous Chronic Disease Care (UCDC) system (self-management)	Improving self-management	UC	Chronic Disease Care

## Footnotes

1 The reason why we focus on six specific conditions is that these conditions share a common risk factor (physical inactivity) and pathogenesis (systemic inflammation). Previous research has shown that interventions targeting specific related conditions, e.g., via risk factors, or with a shared focus on improving function, may be more beneficial than interventions targeting multimorbidity in general (Smith et al., 2016). In addition, these six conditions have been selected as the focus of our overall project (Mobilize- see https://www.mobilize-project.dk/).

2 BCTs range from 1.1 to 16.3 (See Behaviour change taxonomy V1), ✓ means the specific BCT is present

3 UC- Usual Care; UC +- Enhanced Usual Care; CG- Control group; TAU-Treatment as Usual; CBT- Cognitive Behavioural Therapy; EX- Exercise, BAT-Behavioural Activation Therapy; MBAT- Mindfulness Based Art Therapy; MBSR-Mindfulness Based Stress Reduction; MM-Multimorbidity
